# Technical, Ethical, Legal, and Societal Challenges With Digital Twin Systems for the Management of Chronic Diseases in Children and Young People

**DOI:** 10.2196/39698

**Published:** 2022-10-31

**Authors:** David Drummond, Adrien Coulet

**Affiliations:** 1 Department of Paediatric Pulmonology and Allergology University Hospital Necker-Enfants Malades, AP-HP Paris France; 2 Heka Team, Centre de Recherche des Cordeliers Inserm Université Paris Cité Paris France; 3 Centre for Health Informatics University of Manchester Manchester United Kingdom; 4 Inria Paris Research Centre Paris France

**Keywords:** artificial intelligence, pediatrics, medical cyber-physical systems, children, digital twin, child, personalized, cyber-physical, digital health, digital medicine, eHealth, ethics, legal, law, young people, youth, ethical, sensor, monitor, privacy, data collection, paediatric, pediatrician, paediatrician, chronic disease, medical system

## Abstract

Advances in digital medicine now make it possible to use digital twin systems (DTS), which combine (1) extensive patient monitoring through the use of multiple sensors and (2) personalized adaptation of patient care through the use of software. After the artificial pancreas system already operational in children with type 1 diabetes, new DTS could be developed for real-time monitoring and management of children with chronic diseases. Just as providing care for children is a specific discipline—pediatrics—because of their particular characteristics and needs, providing digital care for children also presents particular challenges. This article reviews the technical challenges, mainly related to the problem of data collection in children; the ethical challenges, including the need to preserve the child's place in their care when using DTS; the legal challenges and the dual need to guarantee the safety of DTS for children and to ensure their access to DTS; and the societal challenges, including the needs to maintain human contact and trust between the child and the pediatrician and to limit DTS to specific uses to avoid contributing to a surveillance society and, at another level, to climate change.

## Introduction

Throughout history, the practice of medicine has been constantly impacted by technological advances and societal developments. The first 2 industrial revolutions led to the development of new techniques for obtaining new information about the human body, resulting in the industrial collection of objective and quantitative data in the 20th century, including sensor-based physiological (eg, heart rate, oxygen saturation), biological, imaging, functional test, and increasingly “omics” (eg, genomics, proteomics) data [1]. From the 1970s onwards, the third industrial revolution, also known as the digital revolution, transformed this analogue data into digital data with 2 consequences in the first half of the 21st century: (1) the multiplication of computer models using artificial intelligence (AI) techniques to process patients’ health data and propose a diagnosis, establish a prognosis, and recommend a treatment [1,2] and (2) the possibility of obtaining, using the internet of things (IoT), a comprehensive representation of the patient's health status in real time (ie, a live digital replica of the patient, more commonly known as a “digital twin” [DT]) [3,4]. The combination of AI in DTs could lead to digital twin systems (DTS), in which patients are constantly monitored from their homes and AI techniques adapt their care in real time.

**Figure 1:**
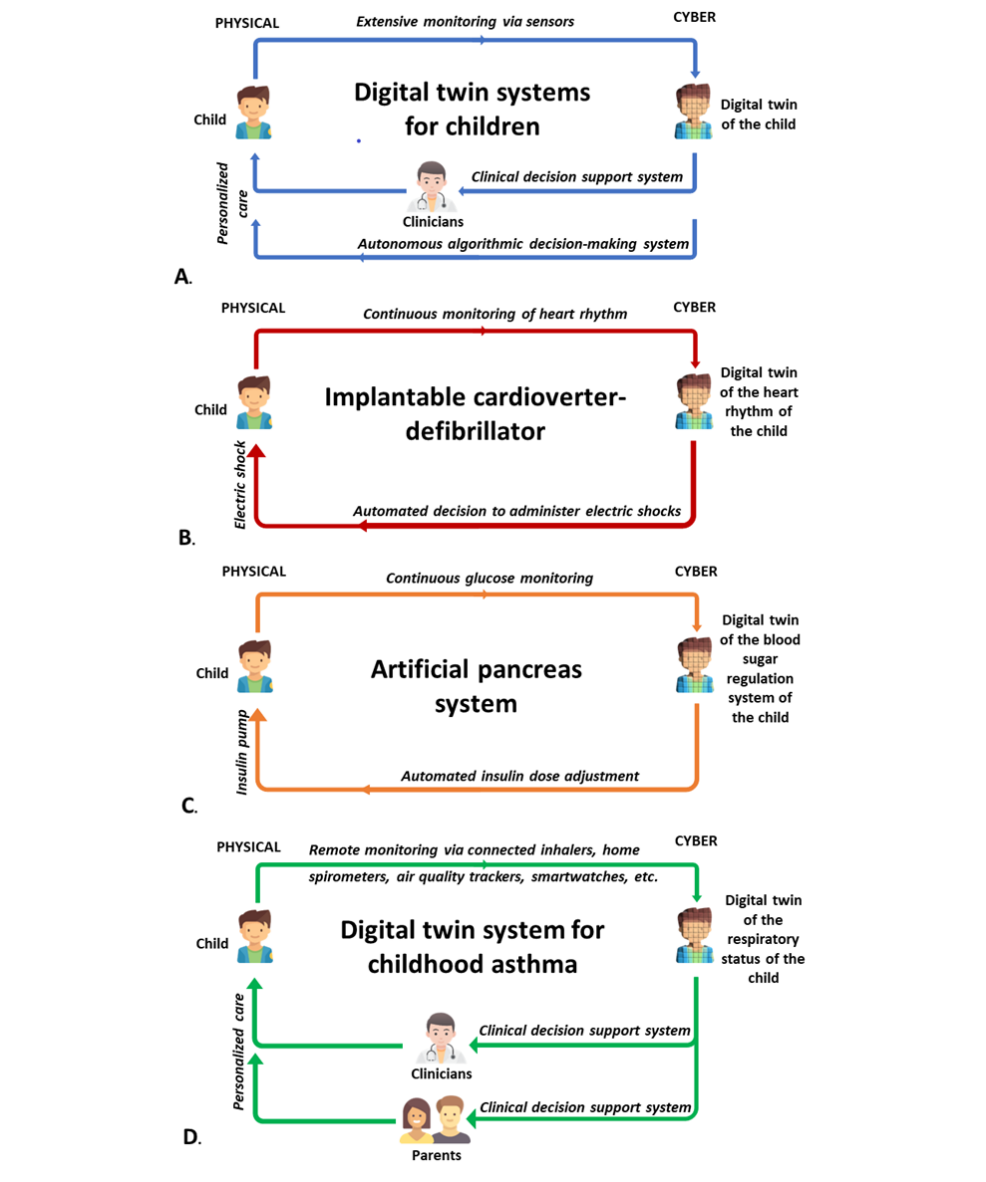
Digital twin systems (DTS) for children and young people with chronic diseases: (A) sensors transform the child's "physical" information into "cyber" or digital data to adapt care, either by a health care professional or autonomously; (B) first example of DTS in childhood, which analyzes the heart rhythm in real time and autonomously delivers electric shocks; (C) more complex DTS, associating continuous monitoring of blood glucose, software adapting the insulin dose, and an insulin pump to deliver this dose; (D) may emerge in the near future for childhood diseases, such as asthma, requiring monitoring of many different determinants, machine learning techniques, and provision of recommendations to different actors (eg, children and their parents, teachers, and health care professionals).

DTS comprise a physical element—the patient—a cyber element—the patient's DT—and 2-way interactions between the physical and cyber elements: Sensors transform the patient's signal into the patient’s DT, and software processes them to act through recommendations to the physician or automated adaptations on the patient's management [2] (Figure 1A). As the human body is extremely complex and its various mechanisms incompletely understood, it is currently not possible to perform a DTS of the whole human body. However, DTS of a function or an organ have gradually appeared. From the 1980s onwards, the first implantable cardioverter-defibrillators appeared: They collect the patient's heart rhythm in real time and, in the event of arrhythmia, automatically deliver an electric shock to restore a normal rhythm (Figure 1B). Such systems can be considered “pre-DTs” in that they originally measured only one parameter (heart rhythm) and the delivery of the electric shock was based on if-else algorithms. With a higher level of complexity, artificial pancreas systems, which combine blood glucose monitors, a virtual representation of the patient's physiology (interactions between measured blood glucose, physical activity, and diet), and actuators (delivery of the predicted optimal insulin dose via the insulin pump), have been developed for children with type 1 diabetes since the 2010s (Figure 1C). These artificial pancreas systems have been shown to improve clinical outcomes and quality of life for patients [5]. In the near future, it is likely that such systems will multiply for other chronic diseases in children, with an even higher degree of complexity. For example, in asthma, the most common chronic disease in children, it is necessary to take into account the multiple determinants of asthma symptoms, including treatment use (emergency and controller treatment) and the environment (eg, pollutants, allergens, weather conditions), to develop different computer models recommending the most appropriate controller treatment in real-time to health care professionals, the most appropriate mitigation measures in case of a high risk of asthma symptoms to families, and a way to involve children and families so that the recommendations made are followed at home. New connected objects (eg, connected inhalers, home spirometers, air quality trackers, smartwatches) and machine learning techniques now make it possible for such DTS to emerge in childhood asthma [6].

The challenges associated with the use of DTS have already been discussed [7-10]. However, just as providing care for CYP is a specific discipline—pediatrics—because of their particular characteristics and needs, providing digital care for CYP also presents particular challenges. Thus, the objective of this article was to review the technical, ethical, legal, and societal challenges associated with DTS for CYP.

## Technical Challenges

The first requirement of digital medicine is digital data. However, both accessing and processing digital data are more complex for CYP than for adults.

### DTS Without Smartphones

Smartphones have a central role in digital health by enabling (1) data collection through built-in functions (eg, GPS for geolocation) and interfaces for patient-reported outcome measures, (2) connection to the IoT via a Bluetooth connection, and (3) patient feedback. In the best-equipped countries, smartphone ownership is limited to 25%, 50%, and 75% of children aged 8 years to 9 years, 10 years to 11 years, and 12 years to 13 years, respectively, complicating the use of digital health interventions for the remote management of children’s diseases [11-14]. Furthermore, it is not intended to promote better smartphone equipment among CYP. In addition to the negative effects of screen time on children's cognitive and socioemotional development, smartphone addiction affects 1 in 4 CYP [15,16]. Thus, even if the DTS interfaces presented on smartphones do not pose an addiction problem, since they will be limited to supporting children in the daily management of their disease, providing a smartphone to children on this occasion could have negative effects on their development due to the other uses they would make of it (eg, games, social media). It is therefore preferable to use specific standalone devices to link the different connected objects as has been done for artificial pancreas systems. In the case of childhood asthma, a smartwatch that would both collect data relevant to monitoring the child's asthma control status (heart rate, oxygen saturation, activity) and provide appropriate and timely recommendations on its screen without allowing other types of internet access would be an interesting solution.

### Designing Devices for Growing CYP

All parents have experienced the recurrent changes in size of clothing and shoes as children grow. A child's height doubles in the first 4 years, from an average of 50 cm to 100 cm, and then increases again by at least 1.5 times over the next 15 years. Similarly, a child's abilities develop impressively, from the infant who does not yet walk and talk to the adolescent capable of the most extreme sports and complex reasoning. Finally, in the medical field, physiological values vary constantly with the age of the child (eg, a heart rate of 50 bpm is normal for a teenager but abnormally low for an infant), as do the expected results of additional tests. This complicates the task for manufacturers of connected devices who need to provide different model sizes, develop appropriate interfaces for children and adolescents of different ages, and adapt the standards for physiological parameters according to the age of the child. These adaptations are not without risk: In the field of cardiac medical devices, an attempt to miniaturize an implantable cardioverter defibrillator resulted in a higher risk of failure in young patients [17]. In the case of an asthma DTS providing recommendations for children, we shall ensure that the recommendations given are age-appropriate, using oral instructions for a 6- or 7-year-old rather than text messages, which would be appropriate for teenagers.

### Protecting Children From Devices

The use of connected objects to collect data from children poses particular risks. Similar to a toy with a defective design, children may choke on, or simply ingest, small parts that may come off the object; they can also be exposed to chemicals that are carcinogenic or mutagenic or to endocrine-disrupting substances. Thus, devices intended to collect data from children’s bodies must be manufactured taking into account the additional risks they may pose to children, which is another challenge compared with manufacturing devices for adults.

### Protecting Devices From Children

In the other direction, children pose specific risks to the connected objects. In the same way that children regularly come home having lost or broken their glasses, connected objects are more likely to be lost or broken when used with children than with adults. This is the consequence of their age-related activities but also of a less cautious attitude toward their personal belongings. For example, connected inhalers that automatically record children's use of asthma medication were reported lost or damaged by up to 50% of families [18,19].

### Developing Models for CYP

CYP pose particular problems when it comes to creating models with traditional supervised approaches from real-world data. The amount of data available is lower, due to the young age of patients (less historical data), logistical and legal difficulties in obtaining the data, and the lower prevalence of diseases in children than in adults in high-income countries [20]. Furthermore, the heterogeneity of the pediatric population is such that it is unsound to learn a single model and necessary to split data in several subgroups. This leads to smaller data sets, which makes it more difficult to obtain performant models for CYP. Fine-tuning models developed for adults may be an option to overcome these limitations, but to our knowledge, it has not yet been applied to create pediatric models.

## Ethical Challenges

**Figure 2:**
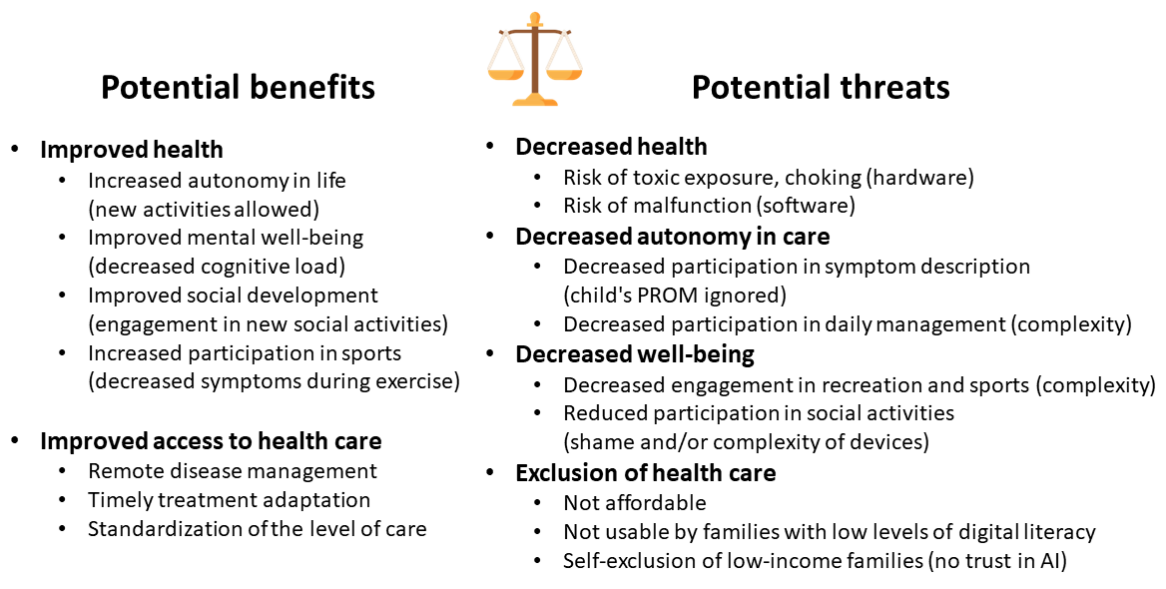
Potential benefits and risks of pediatric digital twin systems (DTS) at the individual level, which require a specific premarket assessment that takes into account not only the health impacts but also the impact of DTS on the child’s physical, mental, or social development. AI: artificial intelligence; PROM: patient-reported outcome measure.

The main ethical question related to the use of digital health in pediatrics is whether the use of DTS is in the best interests of the child. The notion of “best interests of the child” is derived from Article 3 of the United Nations Convention on the Rights of the Child [21]. This is a deliberately ill-defined concept, which needs to be assessed on a case-by-case basis, but several principles that can be used to guide decision-makers have been provided [22]. In this section, we consider how the adoption of digital health for CYP may pose a threat to these “best interests” in light of the 4 principles of biomedical ethics identified by Beauchamp and Childress [23]: respect for autonomy, beneficence, non-maleficence, and justice (Figure 2).

### Preserving the Development of Autonomy

Autonomy means being governed by oneself, in thought and action [24]. As the principle of autonomy cannot be applied as such to children, who are inherently dependent on adults to meet their needs, it was proposed instead to protect the development of the child's autonomy [25]. Pediatricians encourage the development of autonomy in children with diseases by encouraging them to describe their symptoms themselves and by supporting them in taking responsibility for their own care.

#### Preserving Children's Participation in Describing Their Symptoms

Pediatricians encourage children to talk about their symptoms themselves to recognize their place as individuals in the consultation but also because their statements are frequently more accurate than those of their parents [26]. A first risk of DTS is that they do not take into account patient-reported outcome measures from children. Indeed, although it is possible for pediatricians to detect when a child is hesitant in their response or gives a fanciful answer, the direct entry of data by the child into a digital collection system does not allow this assessment. In addition, if the child enters incorrect information, the DTS is likely to provide an incorrect and potentially dangerous action or answer for the child's health. In this context, developers may choose not to include the answers entered by children or ask parents to validate all entries. In both cases, this would be a step backwards in pediatric practice to a time when the child's voice was not taken into account. A second risk of DTS may arise if there is a discrepancy between the child's reported symptoms and the DTS assessment. In this case, adults may tend to believe the DTS rather than the child. Indeed, between 2 contradictory pieces of information, one given by a human and the other by a computer system, humans tend to believe the information given by the computer system [27]. For example, in medicine, clinicians override their own correct decisions in favor of incorrect advice from a decision support system in 6%-11% of cases.[28]. Since children may be perceived as having little credibility [29], this automation bias is likely to be exacerbated in this situation, with the DTS becoming the reference point for adults on the child's condition. For example, consider the case of an 8-year-old asthmatic boy who calls his parents at work to tell them that he is starting to have trouble breathing. The parents check the child's DT status on a dedicated mobile app, note that the risk of an asthma attack is very low, and explain to their child that no, he is not having an asthma attack. How will this child react to the fact that the symptoms he is reporting are not being heard, regardless of the reality or not of the asthma attack? One possibility is that he will lose confidence in himself and his feelings, as a computer system would be more trusted by his parents than his own word.

#### Preserving Children's Empowerment in Their Daily Care

Currently, CYP gradually become responsible for the day-to-day management of their disease, in agreement with their parents and pediatricians. This process can be delayed if DTS are complex to use, whether the complexity is due to the hardware or software components of the DTS. Conversely, DTS can also increase children's autonomy in managing their daily care if they rely on easy-to-use devices and software: In the case of type 1 diabetes, most CYP and parents reported greater control and autonomy in managing their diabetes with insulin pumps after an initial learning period than with injections [30].

### Beneficence

Beneficence refers to the responsibility of professionals to promote the well-being of their patients [31]. It is clear that, before being used in children, DTS must be evaluated for both efficacy and safety through appropriate clinical studies. However, DTS may also pose particular threats to young people and their development.

#### Preserving the Need for Children to Engage in Play and Recreational Activities

By improving their health, DTS are expected to enable children to participate more in play and recreational activities. However, DTS can also prevent children from playing or participating in certain sports: Children using the artificial pancreas system for their type 1 diabetes have reported difficulties when playing sports due to the tendency of the insulin pump to fall [30]. Using a smartwatch for children with asthma may also prevent them from swimming if it has not been anticipated that the device should be waterproof. The right of the child to engage in play and recreational activities appropriate to his or her age is enshrined in the UN Convention on the Rights of the Child (Art. 31), and DTS shall not require children to behave like adults.

#### Preserving Children’s Mental Well-being

Children need to be protected from chronic stress in order to be in the best condition to develop their ability to control their emotions, focus on tasks, and form healthy social relationships [32,33]. DTS can help to reduce this chronic stress through better control of the disease and delegation of decision-making. However, there is a risk that they place the child under continuous pressure. At present, a child with asthma may feel stressed when approaching a doctor's appointment because he knows that he has not taken his medication properly and that his pediatrician is going to re-explain the importance of it. However, in between visits, he is not being monitored and therefore not under the stress of being held accountable. For example, children with asthma may feel under constant pressure with the continuous monitoring introduced by connected inhalers to track their adherence and the associated alerts to their physician if they forget to take their medication [19]. The preservation of the child's physical health must be balanced with their mental well-being.

#### Preserving Children’s Social Development

Children need to interact with their peers, including forming strong friendships, to develop their social skills [34]. Again, DTS can protect these peer interactions by improving the health of CYP and promoting school attendance and participation in various activities. However, DTS can also impair the social life of CYP in 2 ways. First, the complexity of DTS may prevent parents from leaving their child with other adults. In the case of type 1 diabetes, the complexity of the artificial pancreas system and difficulty of explaining how it works were perceived by parents as barriers to leaving children with other people, for example to spend a night at a friend’s house [30]. Second, CYP who have to wear their DTS device on their body feel different from others, which may prevent them from reaching out to others. Adolescent girls with type 1 diabetes expressed how the insulin pump could make them feel different about their bodies and how they tried to hide it for fear of not being accepted by their peers [35]. Similarly, children with a smartwatch or wearable air quality tracker to continuously monitor their asthma could be singled out by their peers.

### Justice

The principle of justice refers to the delivery of equal treatment and care according to the particular patient’s needs, as well as just allocation of available resources [36]. Providing appropriate care for each child according to their needs is all the more important as poor child health limits the potential and development of children, leading to reduced health and life chances in adulthood [37,38]. Yet, the main risk of DTS is that it exacerbates inequalities in health care for children. First, disadvantaged families may not be able to afford DTS, thereby excluding their children from the most effective (if proven) treatment strategies. Second, families with low levels of digital literacy may find it more difficult to use DTS, resulting in less effective treatment adaptations. The third risk is that of self-exclusion of disadvantaged families. Several studies found that lower levels of education, lower levels of employment, and lower household income are associated with negative views and reluctance to participate in research programs involving AI [39-42], raising the risk that algorithms will be trained on data from advantaged families and optimized for these populations. However, DTS can also contribute to reducing inequalities in children's care; for example, by standardizing the care of children and bypassing the doctor for certain decisions, children from disadvantaged families, living in remote areas or with out-of-date doctors, can receive the same care with DTS as children living in privileged areas. For example, the use of connected inhalers that automatically send alerts to health care professionals in case of an asthma attack has been shown to particularly improve the care of the most disadvantaged children [19].

In conclusion, ethical dilemmas can arise in many ways when developing a DTS for CYP, given the need to preserve the health of the child; preserve their physical, mental, and social development; and be anticipated.

## Legal Challenges

As stated by the policy guidance on AI for children from the United Nations International Children's Emergency Fund, children need protection from AI (do no harm) and provision of effective AI systems (do good) [43]. In health, this translates to the need to protect them from the risks posed by DTS and to ensure that children are offered DTS to improve their health.

### Protecting Children From Specific Risks: Do No Harm

Children need special protection from DTS risks for 2 reasons: They have special characteristics that expose them to greater or potentially different risks in comparison with adults, and they are developing beings who may face dramatic consequences for the rest of their lives if their health is adversely affected. Because of these specificities, the approval of a drug for children is subject to a specific process in the United States and European Union. Sponsors are required to provide a pediatric study plan to the Food and Drug Administration (FDA) in the United States or its equivalent pediatric investigation plan to the European Medicines Agency (EMA), which are reviewed by specific committees taking in account pediatric considerations: the FDA's Pediatric Review Committee in the United States and the EMA's Paediatric Committee in the European Union [44].

DTS are not subject to this regulation. Under the current regimes, they are considered medical devices and not medicines. The regulation of medical devices, although recently strengthened in the European Union by the regulation 2017/745 [45], is not as strict as that of medicines from a pediatric perspective [46]. In the United States, the FDA has issued specific guidance for the development of pediatric medical devices [47], but there is no dedicated pediatric committee to assess how these devices are evaluated or adapted for children as there is for medicines and as advocated by the American Academy of Pediatrics [48]. In the European Union, the regulation is limited to stating that the presence of carcinogenic, mutagenic, reprotoxic, or endocrine-disrupting substances must be justified for devices intended for children [45]. Because medical devices are no less risky than medicines, it is important that a similar pathway with pediatric investigation and study plans, reviewed by specialized pediatric committees, is established by the legislators for the approval of pediatric DTS.

### Ensuring CYPs Are Provided With Appropriate DTS: Do Good

If legislators must protect children from the risks that a DTS could pose, they must also protect children from the risk of not having access to DTS that could improve their health. Industry has consistently been less interested in developing drugs for children because of the small number of children affected, increased regulatory constraints, and difficulty of conducting clinical trials in this population, and this trend continues with medical devices including DTS [49-52]. As an example, if multiple algorithms have been used by manufacturers of implantable cardioverter-defibrillators to correctly diagnose atrial and ventricular arrhythmias, these have not been tested in children [17]. For drugs, the situation improved with the introduction of regulations in the European Union and the United States with 2 objectives: (1) to compel pharmaceutical companies to include studies with pediatric populations if the disease or condition for which the drug is indicated occurs in children and (2) to encourage these same companies to carry out these studies by providing a financial incentive in the form of an extension of intellectual property rights [49]. However, such schemes do not exist for medical devices. In the United States, the FDA has launched a series of initiatives to encourage manufacturers to develop pediatric medical devices, with some results [53]. Such initiatives are awaited in Europe.

In conclusion, the risk is that, in the absence of legislative constraints or incentives, market forces will continue to widen the gap between the quantity and quality of DTS developed for adults and children.

## Societal Challenges

**Figure 3:**
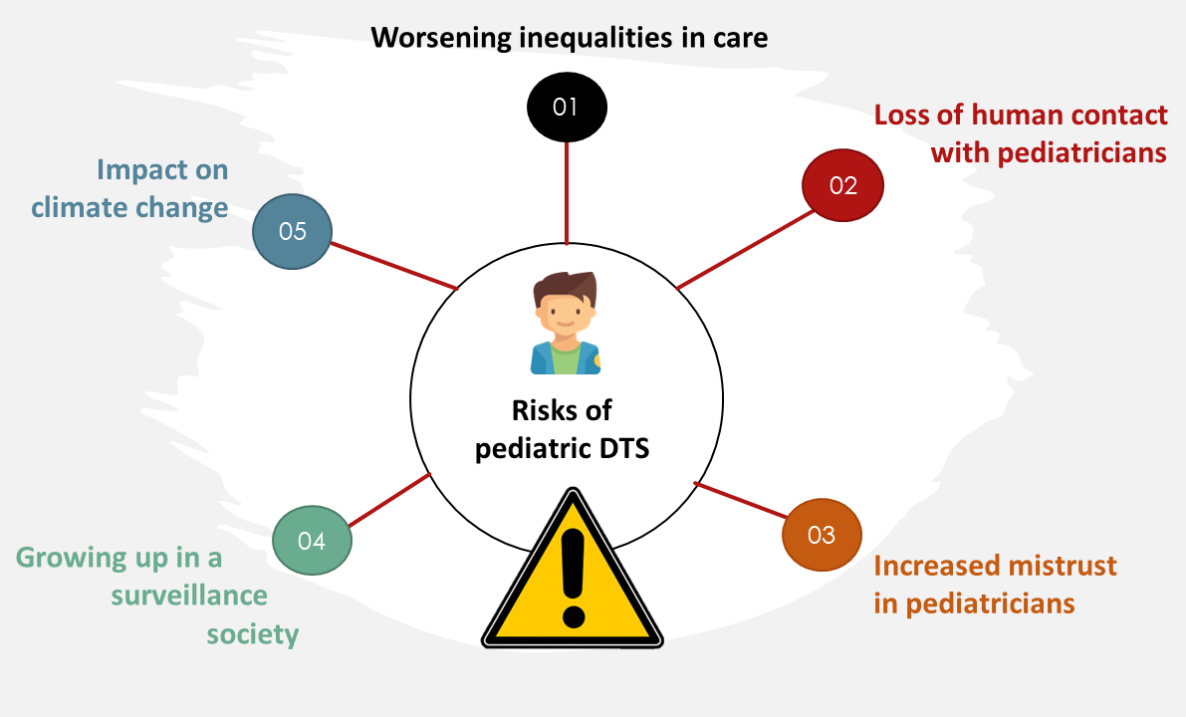
Risks of pediatric digital twin systems (DTS) at the societal level.

DTS in pediatrics is currently limited to a few indications (automated adaptation of insulin doses by artificial pancreas in type 1 diabetes, automated detection and treatment of arrythmia by implantable cardioverter-defibrillators), and pediatricians prescribing DTS are unlikely to feel they are participating in societal change. However, in combination with other digital transformations in society, DTS are likely to lead to profound changes in our lifestyles with important consequences on children. Apart from the risk of exacerbating social inequalities already detailed, DTS can contribute to several societal challenges (Figure 3).

### Preserving Human Contact in Care

If most medical decisions are made autonomously and remotely by DTS, this would result in fewer in-person visits to pediatricians, in line with a more general trend of reduced face-to-face interactions in society [54]. The risk of dehumanization associated with DTS may be a greater problem for pediatricians, who made the choice of a particularly “people-oriented” specialty, in contrast to “technology-oriented” specialties (eg, anesthesiology, radiology) [55]. The risk is that an increasingly technological approach to child health care, combined with a reduction in face-to-face interactions, will lead many to a loss of meaning in practice and to burnout. This may result in a gradual replacement of these doctors by professionals proficient with information technology but less skilled in human relations.

### Preserving Trust

The use of DTS in pediatrics may contribute to a growing distrust of pediatricians. The digitalization of the world, whether through access to medical information on the Internet or conspiracy groups on social networks, has already increased parents' distrust of their pediatricians [56,57]. Families may have less confidence in their pediatrician if they feel that the latter is clearly not mastering the DTS or if the recommendations made by the DTS and the pediatrician differ. In the latter case, explainable models would help the pediatrician to justify disagreement with the DTS and maintain trust. An additional difficulty is that most DTS will aim to prevent a complication or exacerbation from occurring and therefore act while the patient has few or no symptoms. The positive effect of DTS will therefore be less perceptible (absence of an uncertain event) than an exceptional side effect highlighted in social networks. For example, a DTS for childhood asthma may prevent thousands of asthma attacks by providing appropriate recommendations to families when the risk of an asthma attack is predicted for the coming week but be blamed for an undetected severe asthma attack. Finally, pediatricians are perceived to be acting in the best interests of the child, whereas this may not be the case for the companies behind DTS, increasing mistrust [58].

### Preventing Children From Growing Up in a Surveillance Society

The use of DTS requires the collection of a large amount of data, which are increasingly being collected continuously from the child's home or even body. This constant monitoring of children to ensure their safety is a general trend in our societies, in the same way that the proliferation of surveillance cameras was intended to ensure the safety of the population. It can lead to a surveillance society where everyone watches each other, with children being a main target because of their vulnerability. Parents could demand to monitor their children at all times for their safety, since the data are already available, being continuously collected for health purposes. Pediatricians may also ask to use indirect information about parents' behavior collected from the home devices of the child to monitor the risk of child abuse. If such a surveillance society was to emerge, children would grow up in an optimized state of physical health but would probably be more anxious, dependent, and conventional in adulthood, unable to make decisions on their own values [59,60]. Again, legislation implemented by each society will be crucial in ensuring that the best interest of the child is taken into account, at both the individual and societal levels.

### Preserving the Environment

Climate change is one of the major challenges of our century. Young people understand this and are actively campaigning for policies to reduce carbon emissions [61]. CYP are indeed the first to be affected by the consequences of global warming: not only will children born today live in a world with a temperature 4 degrees higher than the pre-industrial era but the effects of global warming on health are much more significant for children than for adults [62]. For example, with regard to the development of diseases favored by global warming, 93% fall upon children [63].

Among the sectors responsible for greenhouse gas emissions, the digital sector is growing in importance every year, increasing in the contribution to global emissions from 2.5% to 3.7% between 2013 and 2019 [64]. Medical devices are the first part of the problem. They usually require raw materials such as rare metals, and most of them are single-use devices with limited recyclability [65]. The flow and storage of the data generated by these devices in data centers are another part of the problem. Currently, data centers account for 1% of total global electricity demand, with about one-half of this energy being used to cool servers [66,67]. Finally, it was recently shown that training a single AI model could generate CO_2_ emissions equivalent to those of a passenger making 300 flights between New York and San Francisco [68]. Thus, even with efforts to reduce electricity consumption in data centers and to move toward “green AI” with efficient models, the general use of DTS would generate a significant amount of greenhouse gas emissions [69]. Conversely, if DTS are effective and improve children's health, they would reduce the use of health care (eg, travel to hospitals, hospital admissions) and in turn would reduce the associated amount of greenhouse gas emissions. Thus, it is currently essential to carry out studies of the environmental impact of digital health interventions, in addition to efficacy trials and medicoeconomic studies, as envisaged by the National Institute for Health and Care Excellence in the United Kingdom [70]. This will participate in safeguarding the future of children.

## Conclusion

**Figure 4:**
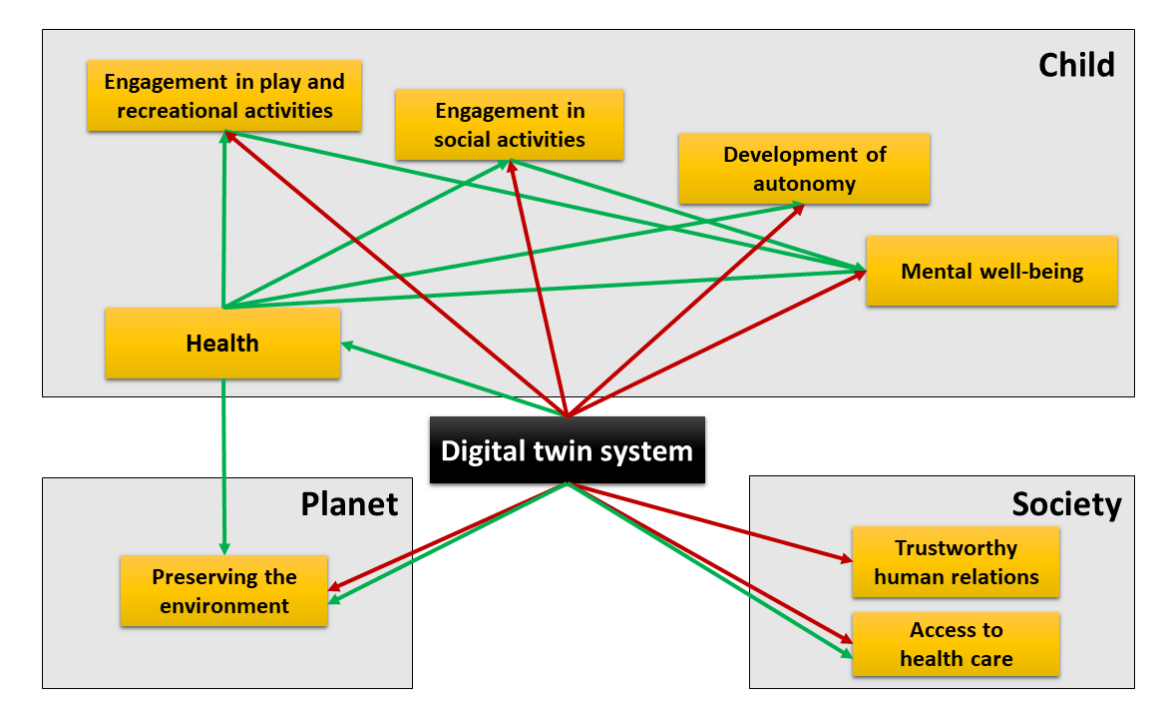
Impact of digital twin systems in pediatrics at different levels and links between the different values. Green arrows indicate a positive impact, and red arrows indicate a negative impact.

The use of DTS for children poses specific challenges at individual and societal levels (Figure 4).

**Figure 5:**
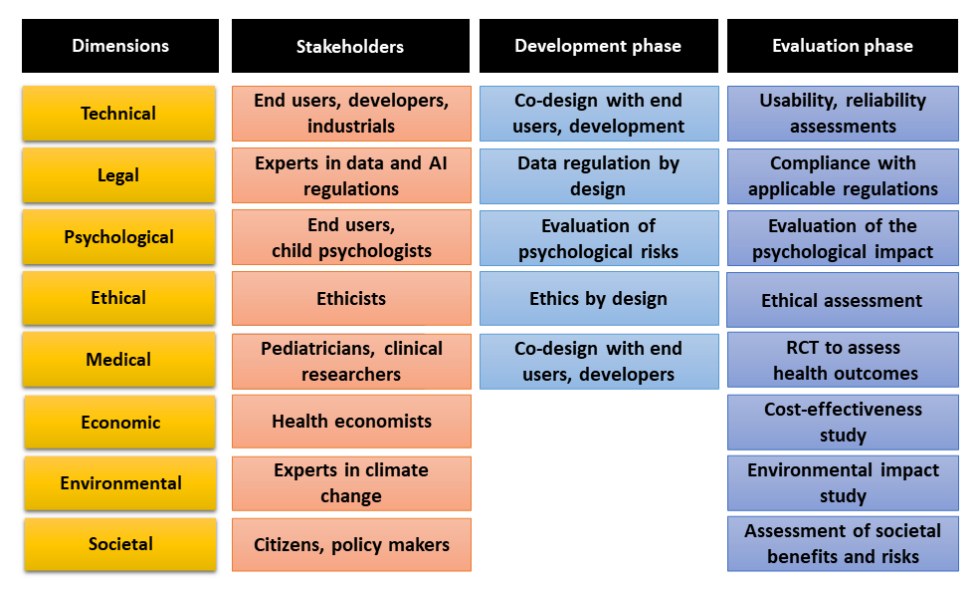
Dimensions of digital twin systems for pediatrics, stakeholders involved, and stakeholders’ roles during the development and evaluation phases. AI: artificial intelligence; RCT: randomized controlled trial.

Since the values at stake are at different levels (preserving the child's life, preserving the child's quality of life and development, preserving life in society, preserving the planet), the ethical approach that seems most appropriate when developing and evaluating a DTS is that of “value pluralism” [71]. This approach recognizes many different, equally fundamental moral values, which may conflict with each other without a predominant value. Indeed, improving children's health is as important as ensuring their quality of life and future development, promoting a society in which they can flourish and leaving them with a livable planet. To take into account all these dimensions, the development of DTS needs to involve many stakeholders at all stages, from the development phase to the evaluation phase (Figure 5).

The evaluation of a pediatric DTS must balance the expected effects on the child's health and its beneficial consequences (increased autonomy, well-being, socialization) against the risks posed by the DTS, whether individual (risk of exposure to toxic substances, stigmatization), societal (contribution to increased inequalities, surveillance society), or global (climate change). This specific evaluation should be supported by specific legislation on pediatric DTS and by incentives by governments and private foundations to promote children's access to DTS. Indeed, children should not be deprived of DTS, which, if effective, could be a real game changer in the management of their diseases.

## Abbreviations

AI: artificial intelligence
CYP: children and young people
DT: digital twin
DTS: digital twin system
EMA: European Medicines Agency
FDA: Food and Drug Administration
IoT: internet of things
